# Diffuse Capillary Spleen Hemangiomatosis: A Rare Cause of Hepatic Dysmorphia

**DOI:** 10.7759/cureus.15320

**Published:** 2021-05-29

**Authors:** Houda Mirali, Imane Kamaoui, Narjisse Aichouni, Siham Nasri, Imane Skiker

**Affiliations:** 1 Radiology, University Hospital Mohammed VI, Oujda, MAR; 2 Radiology, Centre Hospitalier Universitaire Mohammed VI, Oujda, MAR

**Keywords:** hepatic dysmorphia, spleen hemangiomatosis, diffuse, portal hypertention

## Abstract

Spleen hemangiomatosis is the most common type of benign vascular tumor, and it is usually associated with other visceral localizations such as the liver, lymph nodes, skin, and bone marrow. The diffuse form of this condition is defined by the number of locations in one organ. We report the case of a 48-year-old female who sought consultation for left hypochondrium chronic pain. Physical examination subsequently revealed splenomegaly. Imaging showed a diffuse capillary spleen hemangiomatosis, hepatic dysmorphia, and several signs of portal hypertension.

## Introduction

While hemangiomas are the most frequently occurring benign vascular tumor of the spleen, hemangiomatosis is a very rare condition [1] and is characterized by the existence of multiple hemangiomas. Spleen hemangiomatosis is usually associated with other visceral locations such as the liver, skin, bone marrow, and lymph nodes [2]. In this report, we present the case of a very rare isolated spleen hemangiomatosis in a 48-year-old female patient.

## Case presentation

A 48-year-old female with no medical history presented with the complaint of left hypochondrium chronic pain. Physical examination revealed moderate splenomegaly without hepatomegaly. Abdominal ultrasound was performed, which showed heterogeneous splenomegaly measuring 170 mm in the craniocaudal diameter with multiple nodular hyperechoic lesions. The portal vein was dilated, measuring 22 mm, and the liver was dysmorphic with a regular surface. Blood tests including albumin, liver enzymes, bilirubin, and viral hepatitis serology were normal. We completed the exploration with a CT scan. Before contrast iodine injection, we noticed an enlarged spleen, measuring 173 mm in the craniocaudal diameter as well as irregularly lobulated hepatic contour. In the arterial phase, we noticed an enhancement of multiple rounded and nodular lesions of up to 3 cm in size. The splenic artery measured 14 mm in diameter in contrast with the small hepatic, coeliac, and left gastric arteries. These lesions tend to get homogenous in the parenchymal and tardive phases. The portal vein was also enlarged, with portosystemic collateral mapping. No hemangiomatosis was found on the liver or any other visceral area. The patient is currently under medical surveillance. The CT scan follow-up control over the past three consecutive years has revealed the same observations.

**Figure 1:**
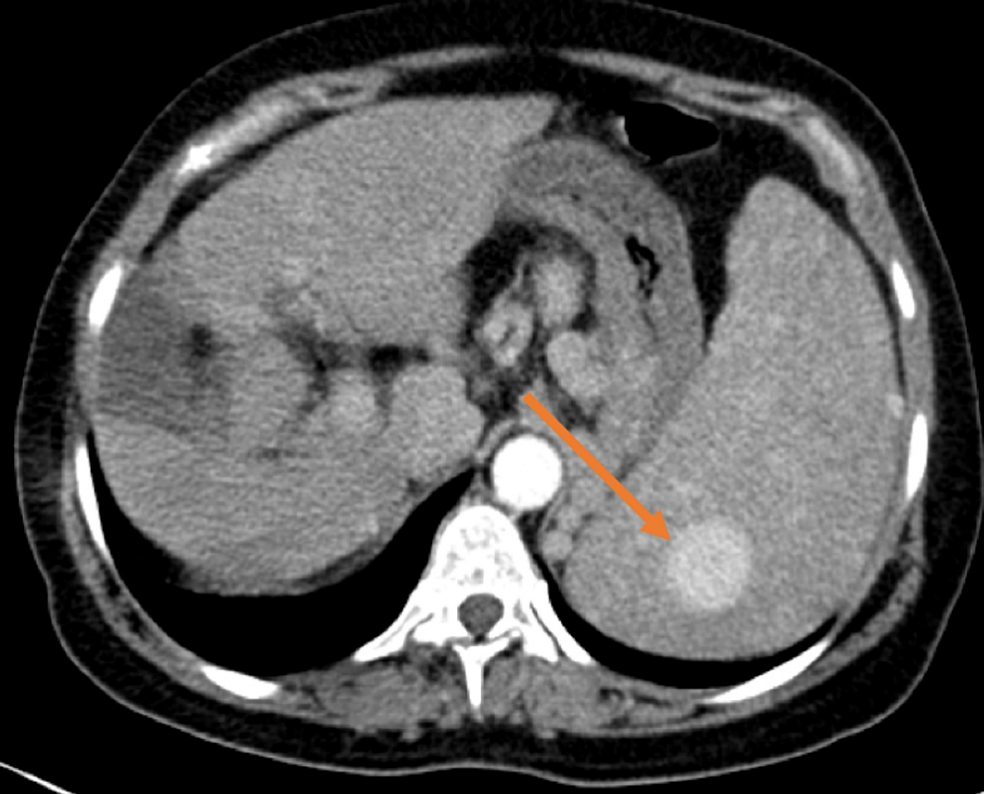
Axial view of abdominal CT scan (arterial phase) The image showed multiple nodular lesions corresponding to hemangiomatosis of the spleen; we also noticed the modifications of the liver contours CT: computed tomography

**Figure 2:**
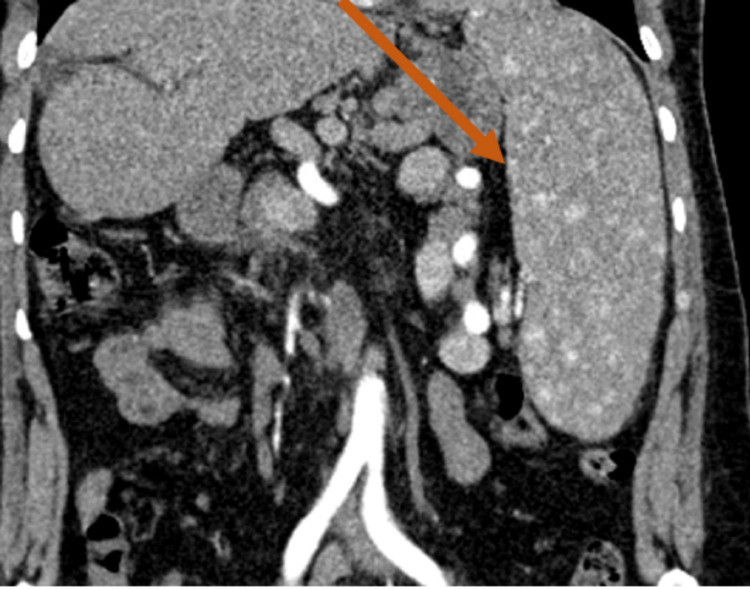
Coronal abdominal CT scan view in arterial phase after iodine injection The image showed the diffuse spleen hemangiomatosis; the enlarged size of the spleen artery was also noticed CT: computed tomography

## Discussion

Isolated spleen hemangiomatosis is a very rare condition secondary to a congenital malformation. Its incidence varies between 0.03-14% based on autopsy findings [3]. This entity predominantly involves arterial capillaries and is associated with a very slow rate of growth. Symptoms or complications usually occur in late adulthood. Splenomegaly is the most frequent symptom of the condition. It is associated with marked enlargement of both splenic artery and vein indicating a high-flow situation. Tada et al. [4] performed a celiac portography on a 39-year-old male diagnosed with splenomegaly secondary to spleen hemangiomatosis, which showed the contrast between a thin hepatic artery and an enlarged splenic artery. The splenic and portal veins were also gigantic, with a similar hyperkinetic situation to arteriovenous communications secondary to diffuse capillary hemangiomatosis of the spleen. In our case, the coeliac and hepatic artery measured around 2 and 4 mm, and the splenic artery and vein were both enlarged, measuring 14 and 20 mm respectively. While portal hypertension most frequently occurs due to hyper-resistance to the flow of blood, in our case it appears to have been caused by increased flow, which would become exclusively hepatopetal. Peri-portal fibrosis of the liver has been reported by many authors [5]. In our case, imaging showed large peri-portal hypodense areas with tardive enhancement after contrast iodine injection and dysmorphic yet regular surface of the liver.

## Conclusions

Spleen hemangiomatosis is a congenital malformation affecting capillary vessels. It is usually asymptomatic and most commonly presents as chronic left hypochondrium pain in late adulthood. In this report, we described a very uncommon situation where spleen hemangiomatosis was associated with portal hypertension and splenomegaly.
